# Seeing in the dark: a metagenomic approach can illuminate the drivers of plant disease

**DOI:** 10.3389/fpls.2024.1405042

**Published:** 2024-07-11

**Authors:** Veronica Roman-Reyna, Sharifa G. Crandall

**Affiliations:** ^1^ Department of Plant Pathology and Environmental Microbiology, The Pennsylvania State University, University Park, PA, United States; ^2^ One Health Microbiome Center, The Pennsylvania State University, University Park, PA, United States

**Keywords:** metagenomics, plant pathogen, workflow, plant diagnostics, novel genes, multi-omics, functional metagenomics, microbiome

## Introduction

There are more microbes on Earth than stars in our galaxy. The Milky Way contains an estimated 100 billion stars and there are around one million trillion trillion (10^30^) Bacteria, Archaea, Fungi, and viruses on our planet, and more are discovered daily (Rappuoli et al., 2023). A leaf contains 10^8^ Bacterial cells in a single gram and magnitudes more, up to 10^12^ Bacteria, may live in the rhizosphere or the space around a plant’s root (Zhang et al., 2017; Dastogeer et al., 2020). Although plants and soils possess a measurable microbial biomass, most microbes cannot be isolated and cultured in a laboratory and therefore are invisible to the naked eye. Similarly, when astronomers calculate the total amount of matter in the universe, a large component cannot be seen by even the most sensitive telescope. Microbes are compared to this undetectable cosmic “dark matter.” Multi-omics tools are a powerful way to “see in the dark” and find microbial communities using their DNA signatures. Here, we focus on metagenomics, the study of genomes from entire microbial communities (microbiomes) that are unculturable and culturable. This approach allows us to find microbes at a deep level of taxonomic resolution and infer predictive physiological functions. A metagenomics approach can be harnessed to detect one-to-thousands of potential pathogens and their relative abundance in a sample. This approach unravels the diversity, structure, and composition of the entire plant-associated microbiome (phytobiome) and the novel genes and metabolic pathways that can cause or suppress plant disease (Crandall et al., 2020).

There is an urgent need to integrate –omics approaches into plant science research. Yet, there are technical challenges that hinder the use of metagenomic methods and tools, particularly during the initial stages of project design. Here, we take a practical view of interdisciplinary tool integration. We (1) discuss questions in plant pathology that could benefit from metagenomics, (2) present best practices to generate metagenomic workflows, 3) critique cutting-edge metagenomic resources, and (4) consider the future of metagenomic research within the plant sciences. Finally, we include a glossary which we hope is useful for those new to metagenomics.

## Where plant disease research meets metagenomics

There is ample room to integrate metagenomics into plant disease research. Plant pathologists study the ecological relationships among virulent microbial pathogens (e.g., Bacteria, Fungi, Oomycetes, nematodes, protists, viruses), susceptible plant hosts, and the environmental factors conducive to disease, commonly referred to as the “disease triangle” (Agrios, 2005). Plant pathology seeks to understand the drivers of disease by detecting potential plant pathogens and host symptoms establishing pathogenicity, describing disease dynamics, and translating the results, when appropriate, to inform disease management. Drawing from a rich and deep training in microbiology, plant pathologists must isolate pathogens from plants and culture them. However, this approach is problematic because most microbes require specific media or host plants for growth, making it impractical to identify suitable growth conditions for thousands of species. Metagenomics and amplicon-based sequencing (synonymous with metabarcoding) are culture-independent methodologies that provide a detailed description of the composition, abundance, and structure of thousands of genomes within a community and can be integrated into the field of plant pathology. Metabarcoding amplifies known gene regions such as 16S, ITS, or 18S which are highly variable in length and GC nucleotide content which allows for finding microbial taxa that differ genetically. In contrast, metagenomics involves sequencing entire microbial genomes, can reveal functional genes that are present, and identify taxa at a fine resolution.

Quantifying the presence, abundance, and functional role of the pathogen(s) are unifying objectives across plant pathosystems. A starting point is using metabarcoding as a tool for disease diagnostics to screen environmental samples (e.g., plant, insect, soil, air, rain) for potential pathogens (Piombo et al., 2021). Based on previous knowledge and peer-reviewed literature, pathogen candidates can be whittled down from a larger list of genera and species. Metagenomics has a higher accuracy for resolving taxa than other detection methods and can recover complete genomes of hard-to-culture microbes. For instance, only 0.5% of Bacteria and Archaea are culturable. Metagenomic tools have been used to identify the cause of citrus huanglongbing, characterize pathogens of rice leaf microbiome, and identify the causal agent of boxwood blight (*Calonectria pseudonaviculata*) (Duan et al., 2009; Yang et al., 2022; Masuda et al., 2024). Pathogens and their pathovars or *formae speciales* (strains that share the same host range) can be found, although this can prove difficult without properly assembled genomes (discussed below). A metagenomic approach allows novel functional gene discovery and finding metabolic pathways that could be involved in pathogenesis such as suppressing host immunity (Aravind et al., 2009; Porras-Alfaro and Bayman, 2011). Moreover, integrating ecological theories into metagenomic studies can deepen our insights into plant diseases (van den Berg et al., 2022). Critical questions that need further research include describing community distributions across different pathosystems, inoculum dispersal across spatial-temporal scales, identifying organisms involved in disease-suppressive soils, and mapping pathogenic traits onto phylogenies to infer potential virulence genes. While established disciplines such as bacteriology, mycology, virology, and epidemiology significantly contribute to our understanding of plant diseases, adopting metagenomic tools offers opportunities to cross-pollinate ideas and knowledge.

## Best practices to generate metagenomic workflows

**Figure 1:**
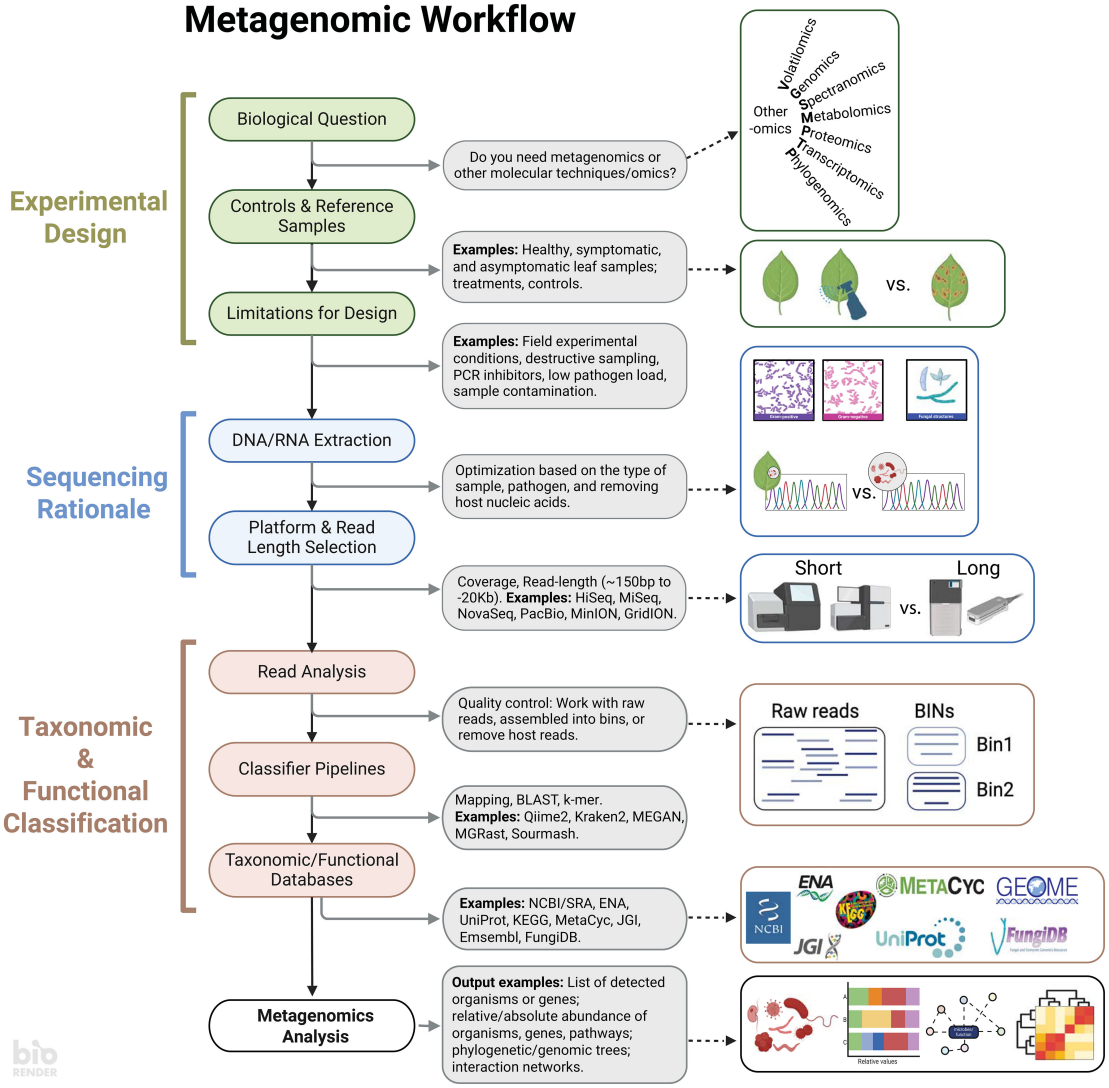
A standard metagenomic workflow can be divided into three main stages: Experimental Design, Sequencing Rationale, and Taxonomy & Functional Classification. Experimental Design covers the biological question of the experiment, as well as the controls and limitations of the system being studied. The specific biological question will help determine whether metagenomics is the best approach, or if other methods such as the study of volatiles, genomes, chemical spectra, metabolites, proteins, expressed genes, or evolutionary history reconstruction might be more appropriate for answering the question. The Sequencing Rationale contains aspects such as nucleic acid extractions and considerations for the sequencing platform. Lastly, the Taxonomy & Functional Classification stage allows to select databases for analyzing the reads. Having a clear understanding of these three stages ensures a clear output to address the research question. Created with BioRender.com.

Important plant disease research questions center around revealing the identity, abundance, and genomic drivers of pathogenesis. To accomplish these questions using -omics, establishing computational workflows can be daunting. Today, the fast pace of such computational-intensive fields has resulted in exponential growth of available workflows. It is challenging to select the most suitable or optimal approach for a specific research question. It is then critical to recognize that metagenomic workflows have distinct components that serve as a foundation for researchers to delineate goals and objectives and enhance research reproducibility. The workflow can be deconstructed broadly into (1) experimental design, (2) sequencing rationale, and (3) classification, each of which is discussed below (**Figure 1**).

## The ingredients of a good experimental design

The initial experimental design should be grounded in specific biological question(s) while considering the inherent limitations of the studied systems (**Figure 1**). Whether exploring unknown microbial communities or assessing treatment effects, a clear understanding of these limitations is crucial for identifying control or reference samples (Ye et al., 2019). A well-defined design determines if a metagenomic approach is necessary for the research or if comparable data from existing microbiome databases can address similar biological questions (Roman-Reyna et al., 2020). It is important to note that metagenomics is not a silver bullet; it cannot elucidate which transcripts and proteins are implicated in pathogenesis, and alternative -omics approaches may be better suited for such inquiries. Additionally, metagenomics cannot prove pathogenicity in the identified taxa; rather, it is a powerful tool to screen for potential communities associated with plant health.

## Sequencing rationale and defining the end goal

A comprehensive experimental design is key for optimizing DNA extractions and sequencing methodologies (**Figure 1**). Inappropriate or poorly optimized extractions resulting in low yield or quality may result in taxa being missed in downstream analyses (Stach et al., 2001). Standardization of extraction protocols is now available and should be reviewed. While budget can dictate the sequencing platform, a clear experimental design facilitates the optimization of sequencing approaches. The number and length of raw DNA reads per sample are contingent on various factors, including the demand for extensive coverage, due to factors such as the plant-to-microbe DNA ratio or microbial diversity. While short-read sequencing is often favored for its high read count and cost-effectiveness, the sequencing strategy depends on whether short or long reads are more informative for extracting the desired information. Short-read platforms offer depth, capturing rare or low-diversity species and providing comprehensive nucleotide diversity information. In contrast, long-read platforms provide better coverage and are especially beneficial for studying microbial functioning (Eisenhofer et al., 2024). Despite the potential for a lower number of reads with long-read platforms, their extended length affords greater selectivity during read classification. Ultimately, the decision between short and long reads hinges on the specific goals and characteristics of the plant-microbe interaction research.

Before sequencing, it is possible to take additional steps to enrich microbial diversity and remove host reads, either with the collected tissue or after DNA extraction. From collected tissue, it is possible to mechanically separate Bacterial cells from plant cells and collect only the Bacterial phase for in-planta Bacterial RNA-Seq (Nobori et al., 2018). After DNA extraction, it is possible to use probes to block the amplification of host sequences that might be similar. Two of the most common methods are peptide nucleic acid (PNA) PCR clamps and C3 spacer (Arenz et al., 2015; Kawasaki and Ryan, 2021). Although these methods are heavily focused on Bacteria (16S rDNA), there are also probes for 18S rDNA that could be adapted for plant research (Liu et al., 2019).

Both long-read and short-read sequencing technologies have their strengths and weaknesses. Advances in long-read sequencing chemistry have reduced error rates, leading to more frequent usage. The choice between long-read and short-read sequencing should be determined by the specific research question. For studies focusing on genome structure, mobile elements, and plasmid movement, long-read sequencing may be more suitable (e.g., to identify plant disease outbreaks (Johnson et al., 2022). On the other hand, short-read sequencing could be more useful for identifying specific alleles or mutations in genes that, for example, affect host recognition or are associated with resistance to biocides. These, and many other technologies now incorporate deep learning and bioinformatic methods to produce high-quality genomes (Zhang et al., 2024).

## Classification using the right database/s for your question

After obtaining raw data, the next step involves classifying it based on taxonomy or metabolic potential (**Figure 1**). Raw reads can be analyzed directly or as assembled contigs, each with its own advantages and drawbacks. Analyzing raw reads directly avoids chimeras but may result in less specific matches due to their small length. Conversely, assembled contigs are more effective in narrowing down hits. The read classification relies on similarity to reference sequences, underscoring the significance of proper databases for finding matches, encompassing organisms or genes of interest. While some software provides pre-built databases for user convenience, these databases may not encompass all relevant organisms, necessitating the creation of custom databases that integrate genomes from private or public collections (Ye et al., 2019) or the use of reference-free detection with machine learning (Johnson et al., 2022). With data and reference in hand, various software options exist for classification (Bolyen et al., 2019; Breitwieser et al., 2019; Pierce et al., 2019; Wood et al., 2019). Many of these tools are open access, with dedicated websites and tutorials for user guidance. Proper experimental design ensures reproducibility and standardization in data analysis workflows.

## Discussion

### Recognizing the limitations of metagenomic resources

**Figure 2:**
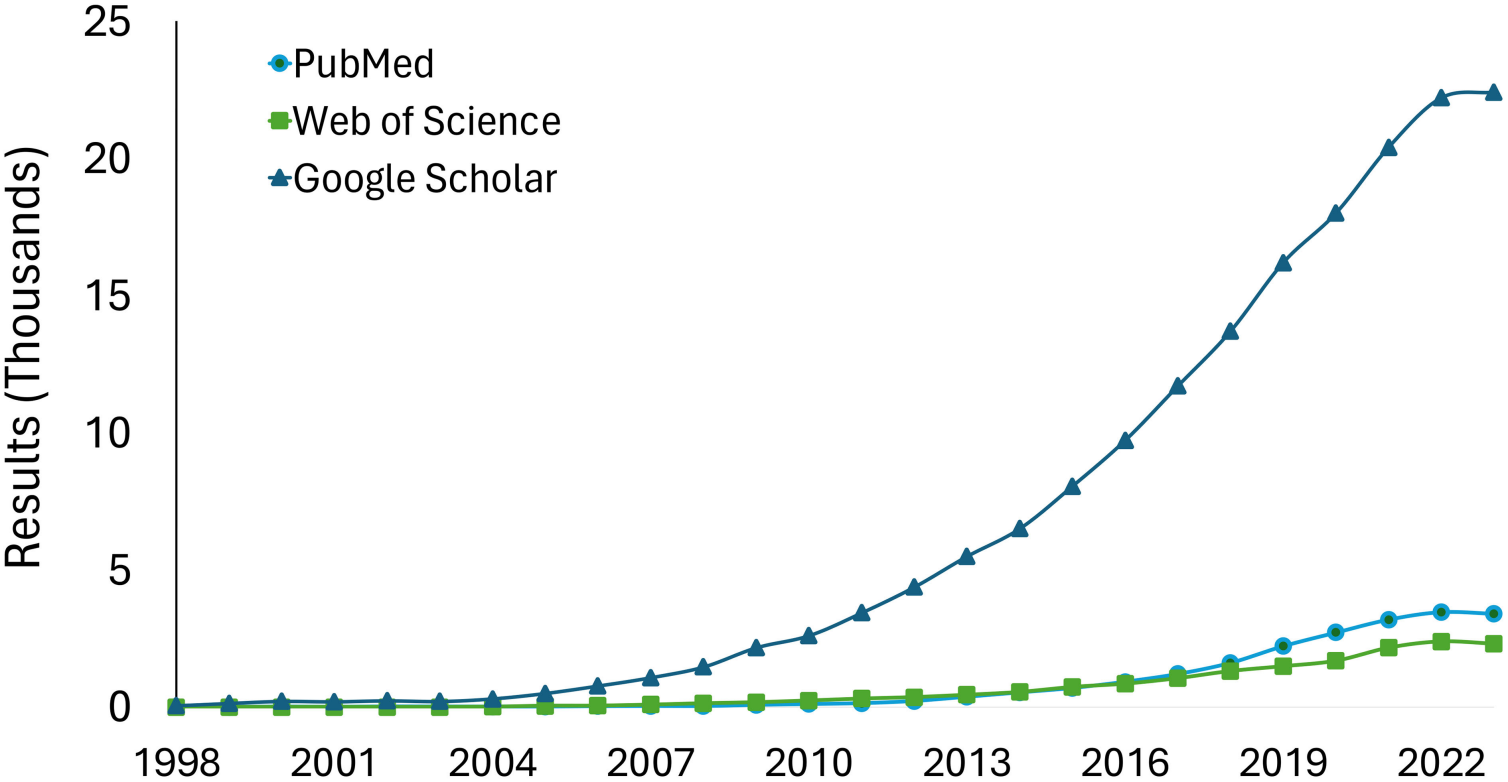
Results in PubMed, Google Scholar, and Web of Science to the query “Metagenomics AND Plants”. The databases were consulted in May 2024 therefore data is included only for complete years.

Metagenomics broadens the scope of research by enabling the exploration of plant-microbial-community questions. The increasing trend in metagenomics studies in plant disease research over time demonstrates its growing adoption and impact in the field (**Figure 2**). However, navigating the realm of metagenomics presents challenges. One major limitation is the high cost of metagenomic sequencing, which makes it difficult to include multiple replicates and adequately cover nucleotide diversity. Additionally, accessibility to software is another hurdle, with disparities in computing resources and internet access among researchers, especially in developing nations (Yek et al., 2022). These multifaceted aspects highlight the ongoing need for strategic planning for effectiveness and accessibility (Carvajal-Yepes et al., 2019). Providing resources (standard protocols) leveraging existing organizations, such as the National Plant Diagnostic Network (NPDN) and the International Plant Diagnostic Network (IPDN), facilitates tool integration.

Reference databases for assigning taxonomic and metabolic pathways, while valuable, have inherent limitations, particularly in their enrichment of viruses, Bacteria, and Archaea, which overshadow data on larger genomes such as Fungi and Oomycetes (Roossinck et al., 2015). Additionally, for nematodes and protists, microscopy may provide more useful for taxonomic identification than -omics approaches since there there is currently a lack of complete genomes available. Enriching databases for these less-explored organisms is essential as microbiome research begins to shift towards more microbe-microbe and functional interactions.

A critical consideration in metagenomics research is the high percentage of environmental DNA associated with dead cells, impacting the accuracy of functional analyses. Understanding this limitation is crucial when associating metagenomic reads with specific functions. One way to overcome this problem is to use fluorescent methods that can distinguish between living and dead cells via amino acid tagging and flow cytometry (Couradeau et al., 2019). Finally, it is crucial to establish standards for using metagenomics as a diagnostic tool. Most studies on Bacterial diseases compare qPCR diagnostic tools with metagenomics results, yet they often overlook the relationship between the number of reads and function or symptoms. An uncertainty is determining the threshold of gene-related reads needed to observe a function. In plant pathology, it remains unclear whether the presence of a pathogen-associated read in asymptomatic samples indicates latent infection or if dominant pathogens are primarily responsible for the disease. Addressing these uncertainties, including eliminating low read abundance species, may result in a loss of information in metagenomic analyses. Metadata, or information about what, where, how, and time of sample collection is critical for reproducibility, and sequences should be uploaded to data repositories such as NCBI and GEOME (Riginos et al., 2020).

### The future of integrative microbiome research

As we envision integrative microbiome research into the future, we believe research in these six topics should be considered to push the frontiers of our knowledge. (i) Plant pathogen evolution in response to environmental pressures. Metagenomics and metabarcoding can reveal shifts in community composition, function, and/or rapid evolution under fungicide, antibiotic, and heavy metal pressure as well as the source of resistance/tolerance genes (Szymanski et al., 2023). (ii) Microbial ecology of pathogens that create and maintain biodiversity. Plant-microbial interactions play a pivotal role in shaping disease dynamics within natural and managed ecosystems. Like predators, pathogens maintain biodiversity through top-down forces (disease and mortality) so that no one species or population dominates or outcompetes others for resources. (iii) Emerging disease, invasive microbes, and global change. Emerging plant diseases are often caused by invasive pathogens or host plants. How and why invasive phytobiomes can cause disease is understudied. (iv) Need for integrating microbiome studies into ecosystem-level experiments. Abiotic factors (e.g., nutrients, moisture) and biotic factors (e.g., plants and microbiomes) drive biogeochemical cycling, linking microbial community growth to plant and microbial metabolic activities. This is essential to understand complex ecological interactions and feedback. Recent research highlights the power of combining metagenomics with other -omics approaches to gain a holistic understanding of plant disease dynamics (Li et al., 2023; Masuda et al., 2024). (v) Encourage a broader spectrum of perspectives and talents in microbiome research. Embracing inclusive microbiome research involves bringing different experiences and backgrounds to the table; this sparks creativity and innovation (Hofstra et al., 2020; Love et al., 2022). Support can be given through workshops, mentorship programs, and opportunities for career growth and advancement. Ensuring equal salaries in academic departments and faculty startups, especially in heavily molecular sectors, is paramount to providing equitable career development, especially for early-career scientists. These strategies cultivate an open and productive environment where underrepresented groups such as women, minorities, people with disabilities, those from resource-limited backgrounds, the queer community, and others can thrive in the plant sciences.

## Author contributions

VR-R: Conceptualization, Writing – original draft, Writing – review & editing. SGC: Conceptualization, Writing – original draft, Writing – review & editing.

## Glossary

**Table:**

*Amplicon*	A short DNA or RNA sequence that is produced via amplification or gene duplication that can be used to find genetic variation in genomes.
*Amplicon-based sequencing*	Also known as metabarcoding. Based on amplifying a known gene region such as 16S, ITS, or 18S to resolve microbiomes and to find multiple taxa in a sample.
*Contigs*	Raw reads are aligned and assembled into longer contiguous sequences. Contigs might be a complete Bacterial chromosome, plasmid, a gene, or a gene fragment.
*Coverage*	Genomic coverage: the percentage of base pairs that will cover regions of interest; gaps that were not filled during sequencing.
*Database*	A structured repository of computational information. File/s with DNA or amino acid sequences that correspond to reference sequences for assigning functions or taxa to fasta files.
*Depth*	How many reads validate the observed sequence; is usually described with an X, for example, 10X coverage.
*k-mer*	DNA sequence of a specific length k that is used for metagenomic read classification.
*Long-reads*	DNA sequence length that can range from 5 to 20Kb and sometimes 1Mb. Help to recover repetitive regions or transposable elements, can get full plasmids as on sequence. Can provide more resolution for taxonomic classification.
*Mapping*	Search matches of raw reads to a reference sequence.
*Metagenome*	Group of genomes, not a gene, from different organisms and viruses.
*Metagenomics*	A technique that uses shotgun sequencing to obtain sequences of all extracted DNA. Shotgun refers to DNA that has been fragmented before sequencing.
*Microbiome studies*	Research on the relationships of microorganisms and communities in an environment drawing from microbial ecology.
*Pathosystems*	This can be a single or multiple interactions and dynamics between: plant host/s, virulent pathogen/s, and environment/s conducive to plant disease.
*Short reads*	DNA that ranges from 70-300bp. A sequencing file can have millions to billions of reads. Most are used to capture differences at the nucleotide level and recover low abundant reads.
